# Daptomycin-Induced Acute Liver Failure: A Rare Case Report

**DOI:** 10.7759/cureus.46692

**Published:** 2023-10-08

**Authors:** Medha Rajamanuri, Julton Tomanguillo, Mark Ayoub, Nadeem Anwar

**Affiliations:** 1 Internal Medicine, Southern Illinois University School of Medicine, Springfield, USA; 2 Internal Medicine, Charleston Area Medical Center, Charleston, USA; 3 Gastroenterology and Hepatology, Charleston Area Medical Center, Charleston, USA

**Keywords:** antibiotic side effects, acute liver injury, drug induced liver failure, acute liver failure, daptomycin

## Abstract

Acute liver failure (ALF) is characterized by severe liver injury, encephalopathy, and impaired coagulation/synthetic function. Drug-induced liver injury (DILI) can rarely, in a dose-dependent manner, lead to ALF. This article presents a rare case of daptomycin-induced acute liver failure in a patient with no prior liver disease. A 73-year-old male with multiple comorbidities including heart failure, diabetes, and chronic kidney disease received daptomycin treatment for diabetic left foot osteomyelitis. Five days after starting therapy, he developed weakness, jaundice, and drowsiness, leading to ICU admission. Physical examination and labs revealed hepatomegaly, elevated liver enzymes and abnormal ultrasound findings. Autoimmune and infectious causes were ruled out. Daptomycin was discontinued, and the patient’s labs showed significant improvement within three days. One week after recovery from acute liver failure, he experienced cardiogenic shock due to worsening of his underlying heart failure and was transferred to the Cardiac ICU before ultimately being discharged to inpatient hospice care. To our best knowledge, this is the first reported case of daptomycin-induced acute liver failure, presenting with massive liver enzyme elevations, synthetic dysfunction, and encephalopathy. The Naranjo scale score suggests a probable causal relationship between daptomycin and liver injury. While a few cases of daptomycin-induced liver injury have been reported, there are no previous reports of acute liver failure. The rapid development of liver failure following daptomycin administration and subsequent recovery after discontinuation is noteworthy. However, various confounding factors and the mechanism of daptomycin-induced liver failure remain unclear. Further research is needed to identify predisposing factors and better understand this rare complication. While rare, this care also raises caution to follow liver function closely while prescribing daptomycin.

## Introduction

Acute liver failure (ALF) is characterized by severe acute liver injury (onset <26 weeks) with elevated transaminases, encephalopathy, and impaired coagulability/synthetic function (international normalised ratio (INR) of ≥1.5) in a patient without prior liver disease or cirrhosis [1]. Adverse drug reactions leading to acute liver injury are underreported and underestimated. The most common causative drugs include acetaminophen, antibiotics (notably amoxicillin-clavulanate), anti-tuberculosis medications, anticonvulsants and herbal supplements [2]. Drug-induced liver injury (DILI) causing hepatic failure is rare and has mostly been reported in a dose-dependent manner with acetaminophen [3]. The common side effects of daptomycin therapy are headache, dizziness, restlessness, and diarrhea. In higher doses, it has been shown to cause myositis and rhabdomyolysis in combination with statins. Current literature suggests that about 2% to 6% of patients on daptomycin therapy experience elevated liver enzymes which are reported to be mild to moderate and usually self-limiting without the need for discontinuation of the drug [4]. As per the reputed liver tox website, a likelihood score of C has been assigned to daptomycin which indicates probable cause of clinically relevant liver injury [4]. However, we present a rare case of daptomycin-induced acute liver failure in a patient with previously normal liver function.

## Case presentation

A 73-year-old male with a past medical history of type 2 diabetes mellitus (T2DM) with neuropathy, stage 3 chronic kidney disease (CKD), coronary artery disease (CAD), restless leg syndrome, heart failure with reduced ejection fraction (HFrEF) (EF = 10-15%), non-alcoholic fatty liver disease, benign prostatic hyperplasia (BPH), atrial fibrillation, and antineutrophilic cytoplasmic antibody (ANCA) vasculitis (Wegener’s) was admitted with diabetic left foot osteomyelitis. He underwent a left great toe amputation. Surgical cultures showed methicillin-resistant Staphylococcus aureus (MRSA) growth, and he was started on daptomycin 375mg intravenous (IV) daily. About five days after initiating daptomycin therapy (as per antibiotic sensitivity) he began to experience weakness, jaundice, and drowsiness, leading to his admission to the ICU. 

During the physical examination, he displayed asterixis, diffuse abdominal tenderness, and somnolence. His liver enzymes were significantly elevated: aspartate transaminase (AST) 2311, alanine transaminase (ALT) 5047, prothrombin time (PT) 57, INR 9.3, and total bilirubin 3.5, in comparison to his admission levels of AST 24, ALT 31, and total bilirubin 0.8 (Table 1). Other relevant lab values include white blood cells (WBC) 9200, blood urea nitrogen/creatinine (BUN-Cr) 67/1.9. Abdominal ultrasound revealed hepatomegaly with a liver span of 18.1 cm, along with a small amount of perihepatic fluid but no evidence of steatosis, cirrhosis or masses was noted. The gallbladder appeared without stones or sludge. Tests for autoimmune and infectious causes of acute liver injury, including antinuclear antibodies (ANA), anti-mitochondrial antibodies, smooth muscle antibodies, and a viral hepatitis panel (including hepatitis E, immunoglobulin M (IgM) anti-hepatitis A virus (HAV), IgM anti-hepatitis B surface antibody (anti-HBs) or hepatitis B surface antigen (HBsAg), and anti-hepatitis C (HCV) or HCV RNA), were performed and all were negative. Risk factors for other forms of acute liver disease, such as alcohol use, recent weight gain, signs of decompensated heart failure, sepsis (qSOFA-1), hypoxemia, or hypotension (<90/60), were ruled out. Hepatic duplex results were normal. 

**Table 1:** Laboratory values and timeline ALT: Alanine transaminase, ALP: Alkaline phosphatase, INR: International normalized ratio

Time	Symptoms	ALT (U/L)	Alk P (U/L)	Bilirubin (mg/dL)	INR	Other
0	Alert, oriented	31	120	0.8	1.5	Admission
5 days	Confused, Icterus	3211	203	3.4	6.2	Daptomycin discontinued
7 days	worsening somnolence, asterixis+	5047	359	3.5	9.3	Peak of liver enzymes
10 days	Improving mentation	1209	286	2.7	7.4	Five days after discontinuation of daptomycin
17 days	New symptoms of worsening shortness of breath, somnolence	326	154	1.5	2.6	Heart failure exacerbation
Normal Values		<40	<130	<1.2	1.1	

Daptomycin was discontinued on day seven, and he was started on n-acetyl cysteine (NAC) therapy, leading to gradual improvement in his mental state. His lab results began to trend downward within two days of discontinuing the drug. He was subsequently transferred out of the ICU. One week after the transfer, he developed cardiogenic shock and was initiated on dobutamine and norepinephrine. It was determined that his shock was secondary to bi-ventricular heart failure vs septic shock in the setting of negative blood culture and normal WBC count, leading to his admission to the Cardiac ICU service. However, despite the improvement in his liver function tests (LFTs), he was unable to be weaned off inotropic support. After continued discussion with his family regarding goals of care, the decision was ultimately made to discharge the patient to inpatient hospice (Table 2).

**Table 2:** Key information

Medication:	Daptomycin IV (325 mg daily)
Pattern:	Hepatocellular
Latency:	onset in two to three days, peaked at seven days
Recovery:	Liver enzymes began down-trending at three days of discontinuation of daptomycin (discontinued on Day 5 of hospitalization)
Other medications:	Eliquis, gabapentin, lisinopril, ferrous sulfate, furosemide, tamsulosin, melatonin
Comorbidities	Type 2 diabetes mellitus (T2DM) with neuropathy, stage 3 chronic kidney disease (CKD), coronary artery disease (CAD), non-alcoholic fatty liver, restless leg syndrome, heart failure with reduced ejection fraction (HFrEF) (EF = 10-15%), benign prostatic hyperplasia (BPH), atrial fibrillation, and antineutrophilic cytoplasmic antibody (ANCA) vasculitis (Wegener’s) was admitted with diabetic left foot osteomyelitis

## Discussion

To the best of our knowledge, this represents the first documented case of daptomycin-induced acute liver failure, characterized by significant elevations in LFTs, impaired synthetic function, and encephalopathy [4]. Concurrent acute-on-chronic renal failure is observed, but rhabdomyolysis is absent [5]. After thorough exclusion of other potential causes of ALF, daptomycin was identified as the likely instigator. The Naranjo scale (The adverse drug reaction probability scale), one of the instruments employed to establish causality in DILI cases, assigns a score of 5 to this patient (scores vary from -4 to +13; drug reaction is considered definite if the score is >/=9, probable if 5-8, possible if 1-4, and doubtful if </=0)[6], indicating daptomycin as the probable cause of the liver injury [7].

Historically, only a handful of case reports have hinted at DILI associated with daptomycin leading to elevated LFTs in the absence of rhabdomyolysis. For instance, Janda et al. chronicled a case where elevated transaminase levels manifested seven days post daptomycin administration, reaching their zenith on the 10th day (AST/ALT/gamma-glutamyl transferase (GGT)/alkaline phosphatase (ALP)/creatine kinase (CK): 225/158/1022/723/140 U/L, respectively). Liver enzyme levels normalized within nine days following daptomycin cessation [8]. Another report by Bohn et al. featured a patient who exhibited asymptomatic transaminitis and increased bilirubin after daptomycin treatment, without concomitant organ dysfunction or rhabdomyolysis. Daptomycin was identified as the cause of liver injury after excluding alternative etiologies [9]. Notably, neither of these cases demonstrated abnormalities in synthetic function or encephalopathy, a contrast to our index case.

Abraham et al. documented a daptomycin-associated instance of acute liver and renal injury in the absence of elevated CK levels and rhabdomyolysis. The patient, undergoing treatment for MRSA osteomyelitis, exhibited AST/ALT/serum creatinine levels of 6020/8050/2.9 after five weeks of treatment. Additionally, elevated ALP (370) and total bilirubin of 2.0 mg/dl were observed, while CK levels remained within normal ranges. Following daptomycin cessation, liver enzyme and serum creatinine levels normalized six weeks later, implying DILI attributed to daptomycin [10]. From published evidence usually the latency to onset was five weeks, and resolution was slow with mild abnormalities still present six weeks later. Thus, clinically apparent acute liver injury which progressed to acute liver failure from daptomycin in our case is unique.

## Conclusions

In conclusion, while it is interesting to note the acuity/rapidity of development of liver failure following daptomycin administration and recovery after discontinuing the drug, there could be several confounding factors like underlying comorbidities, other medications leading to such severity, and we are uncertain of the mechanism by which daptomycin causes liver failure. Further research is warranted to determine the factors predisposing the patients to daptomycin-induced liver injury progressing to liver failure. It is very relevant as daptomycin is frequently used, and clinicians need to closely monitor liver function monitoring especially in patients with known liver disease. 
